# Effective recovery of highly purified CD326^+^ tumor cells from lavage fluid of patients treated with a novel cell-free and concentrated ascites reinfusion therapy (KM-CART)

**DOI:** 10.1186/s40064-015-1508-3

**Published:** 2015-12-17

**Authors:** Yukino Kimura, Yui Harada, Noriko Yasuda, Takefumi Ishidao, Seiichi Yusa, Keisuke Matsusaki, Yoshikazu Yonemitsu

**Affiliations:** R&D Laboratory for Innovative Biotherapeutics, Graduate School of Pharmaceutical Sciences, Kyushu University, 6 floor, Collaborative Research Station I, 3-1-1 Maidashi, Higashi-ku, Fukuoka, 812-8582 Japan; Tella, Inc., Tokyo, Japan; AllGene, Inc., Kanagawa, Japan; Ascites Treatment Center, Kanamecho Hospital, Tokyo, Japan

**Keywords:** CART, Tumor lysate, Ascites

## Abstract

For the production of tumor-specific vaccines, including dendritic cell (DC) vaccines, the tumor cells themselves are an ideal source. Floating tumor cells in the ascites fluid from patients with malignant ascites are a good candidate source, but it is not easy to obtain pure tumor cells from ascites because of various types of cell contamination as well as protein aggregates. We here report an effective method to recover pure tumor cells from malignant ascites. We used lavage fluid from 13 patients with malignant ascites who were treated with modified cell-free and concentrated ascites reinfusion therapy (KM-CART). Cellular components were separated from the lavage fluid by centrifugation, enzymatic digestion and hemolysis. Tumor cells were purified by depleting CD45^+^ leukocytes with antibody-conjugated magnetic beads. The tumor cell lysate was extracted by freeze-and-thaw cycles. The mean obtained total cell number was 7.50 × 10^7^ cells (range 4.40 × 10^6^–2.48 × 10^8^ cells). From this fraction, 6.39 × 10^6^ (range 3.23 × 10^5^–2.53 × 10^7^) CD45^−^ cells were collected, and the tumor cell purity was over 80 % defined as CD45^−^CD326^+^. A sufficient amount of tumor lysate, average  = 2416 μg (range 25–8743 μg), was extracted from CD45^−^CD326^+^ tumor cells. We here established an effective method to produce highly purified tumor cells from KM-CART lavage fluid. The clinical feasibility of this simple preparation method for generating tumor lysate should be examined in clinical studies of DC vaccines.

## Background

Cancer vaccines targeting tumor-specific antigens, including dendritic cell (DC) vaccines, are a promising alternative against malignancies. Dendritic cells are unique antigen-presenting cells (APCs) that can stimulate innate and acquired immune responses. We have conducted several clinical trials in which we observed no serious adverse events related to DC vaccination (Takahashi et al. 2013; Kobayashi et al. 2013, 2014a, b). We also found that the combination therapy of DC vaccines and an activated T cell infusion could be an effective treatment for patients with pancreatic cancer (Kimura et al. 2012). For antigen-loaded DCs, synthetic peptides can be prepared uniformly and according to the expression patterns on tumor tissue, but it has been difficult to adapt such peptides to tumor antigenic mutation. Moreover, synthetic peptides are restricted by the HLA of patients.

Alternatively, the autologous tumor lysate could have the following advantages: (a) it could provide multiple antigenic epitopes for recognition by specific T cells. Tumors are heterogeneous cell populations among individuals, and they contain unidentified tumor-associated antigens (TAAs). (b) Targeting multiple antigens could be advantageous and may eliminate the need for the immune selection of single-antigen-loss tumor variants (Fields et al. 1998; Shimizu et al. 1999). Thus, tumor cells in the ascites from patients are a good candidate source for tumor-specific vaccines.

Malignant ascites (MA) is defined by the US National Cancer Institute (NCI) as “a condition in which fluid containing tumor cells collects in the abdomen” and it represents an advanced state of ovarian, pancreaticobiliary, or gastrointestinal cancer (Chung and Kozuch 2008). Malignant ascites causes severe abdominal distension, dyspnea and loss of appetite, and it can thus result in reduced activities of daily living and quality of life. Cell-free and concentrated ascites reinfusion therapy (CART) is a therapeutic method in which the ascitic fluid or pleural fluid is collected from a patient with ascites, and then cellular components including bacteria and leukocytes are removed by filtration. The conventional CART system uses a complicated circuit and requires handling via a special pump system. Furthermore, it has an unsatisfactory filtration capacity with the membrane becoming clogged after filtration of about 2 L of cancerous ascites. On the other hand, the previously developed KM-CART system has a simpler circuit and employs an external pressure system with a membrane-cleaning function that can collect filtrated cells from lavage fluid (Matsusaki et al. 2011a). Thus, in the present study, we attempted to recover purified tumor cells from KM-CART lavage fluid and to obtain the cell lysate for DC vaccines.

## Methods

### Patients

The collection and analyses of KM-CART lavage fluid from 13 cancer patients were done with written informed consent under the approval of the Institutional Review Board of Kyushu University. The characteristics of the patients are summarized in Table 1.

**Table 1:** Patients characteristics

Gender (M/F)	3/10
Age (years)	57.0^a^ (35–91)
Location of original tumor	
Ovary	6
Stomach	3
Gallbladder	1
Colon	2
Unknown	1
Median amount of removed ascites (mL)	5900^a^ (2800–11,500)
Median amount of lavage fluid (mL)	1700^a^ (800–6800)

^a^Median (range)

### Cell preparation

KM-CART was conducted as described (Matsusaki et al. 2011b). KM-CART lavage fluid was kept at 4 °C until use (for 2–3 days). The cellular fraction was separated by centrifugation at 1500*g* for 5 min and washed once with RPMI-1640. Minced large clusters were digested by collagenase (50 μg/mL, Nacalai Tesque, Kyoto, Japan) and DNase I (100 μg/mL, Roche Diagnostics, Basel, Switzerland). The erythrocytes were then burst in pure water for 60 s, and the remaining cells were washed with polymyxin B solution (50,000 U/mL, Sigma-Aldrich, St. Louis, MO) and passed through 100 μm nylon cell strainer (CORNING, New York, NY). For tumor cell enrichment, CD45^+^ cells were depleted with CD45-MicroBeads (Miltenyi Biotec, Bergisch Gladbach, Germany) according to the manufacturer’s instructions.

### Flow cytometric analysis

The cells from KM-CART lavage fluid were stained with the following FITC-, PE-, or PerCP-Cy5.5-conjugated monoclonal antibodies (mAbs): CD3, CD4, CD8, CD11b, CD19, CD25, CD33, CD45, and CD326 (BioLegend, San Diego, CA). The appropriate conjugated isotype-matched IgGs were used as controls. The cells were analyzed using a FACSCalibur and CellQuest software (Becton–Dickinson, Lincoln Park, NY). The final data were prepared with FlowJo 7.6 software (Tree Star, Ashland, OR).

### Wright–Giemsa staining

The cells from KM-CART lavage fluid were morphologically assessed using the Wright–Giemsa staining method. Cells were prepared on slides by a Cytospin (Shandon Southern, UK) and stained with Wright–Giemsa stain. The cell morphology was examined under a light microscope, BZ-9000 (KEYENCE, Osaka, Japan).

### Tumor cell lysate preparation

CD45-depleted cells were re-suspended in D-PBS(−) (Wako, Japan) and lysed by five freeze (in liquid N_2_)/thaw (in 37 °C water bath) cycles followed by centrifugation. Lysate was centrifuged at 15,000*g* for 30 min at 4 °C. The supernatant was recovered and the protein content was measured with a NanoDrop 2000 spectrophotometer (Thermo Fisher Scientific, Waltham, MA).

### Statistical analyses

All data are expressed as the mean ± SEM. The data were examined using the Mann–Whitney U-test.

## Results

**Fig. 1:**
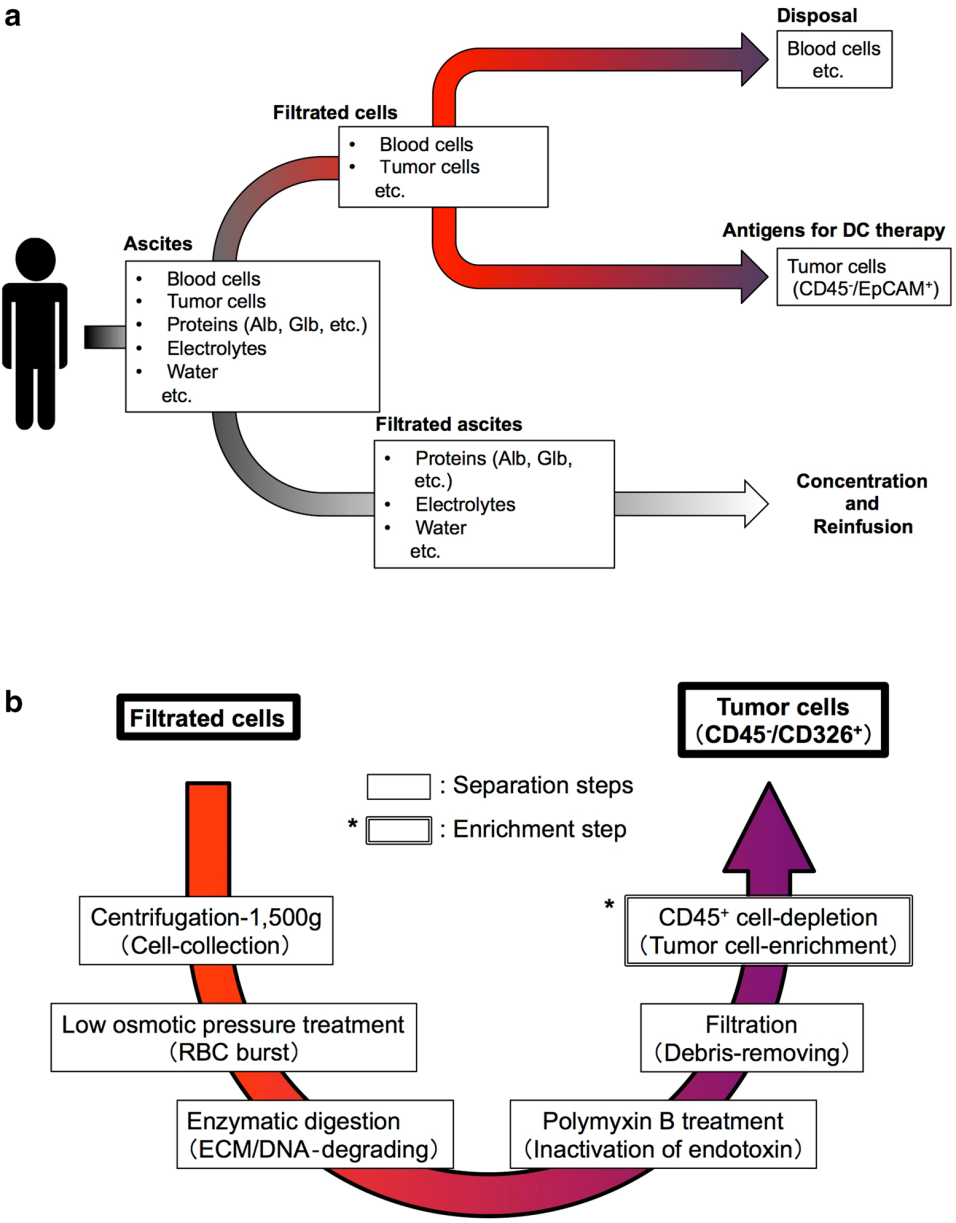
Schematic diagrams of the KM-CART tumor cell-recovery system. Cellular components (blood cells, tumor cells, etc.) are collected by the KM-CART system, and then the separation and enrichment of tumor cells (CD45^−^/EpCAM^+^) is performed. **a** Processing of ascites. **b** Processing of lavage fluid

Schematic diagrams of the tumor cell-recovery system are shown in Fig. 1. Filtrated cells from the lavage fluid of CART for cancerous ascites were collected from 13 subjects with various types of malignancies (Table 1).

**Fig. 2:**
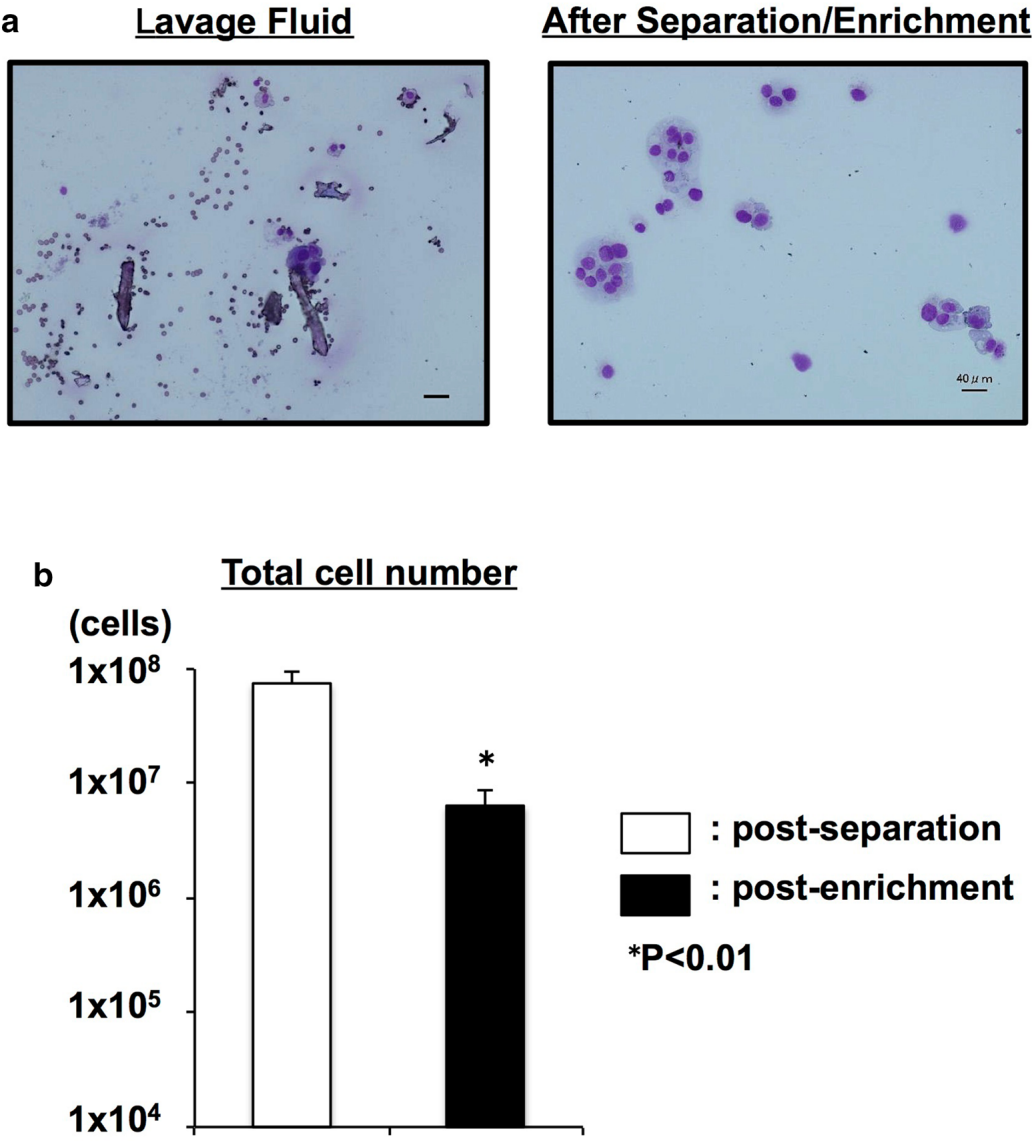
Collection of cell components from lavage fluid. Total cellular components were separated from KM-CART lavage fluid by centrifugation, enzymatic digestion, RBC-burst, polymyxin B treatment and filtration (separation steps) followed by CD45^−^ depletion (enrichment step). **a** Wright–Giemsa-stained cytocentrifuge preparations are shown. *Bars* 40 μm. **b** The mean obtained total cell numbers. After the separation steps: 7.50 × 10^7^ cells, and after the enrichment step: 6.39 × 10^6^ cells

Typical clusters of tumor cells and white blood cells (WBCs) were observed in the lavage fluid, and tumor cells were highly enriched after separation/enrichment (Fig. 2a). After the separation steps (centrifugation, enzymatic digestion, red blood cell (RBC)-burst, polymyxin B treatment and filtration), the mean obtained total cell number was 7.50 × 10^7^ cells (range 4.40 × 10^6^–2.48 × 10^8^ cells). After the enrichment step (after CD45-depletion), the mean obtained total cell number was 6.39 × 10^6^ cells (range 3.23 × 10^5^–2.53 × 10^7^ cells) (Fig. 2b).

**Fig. 3:**
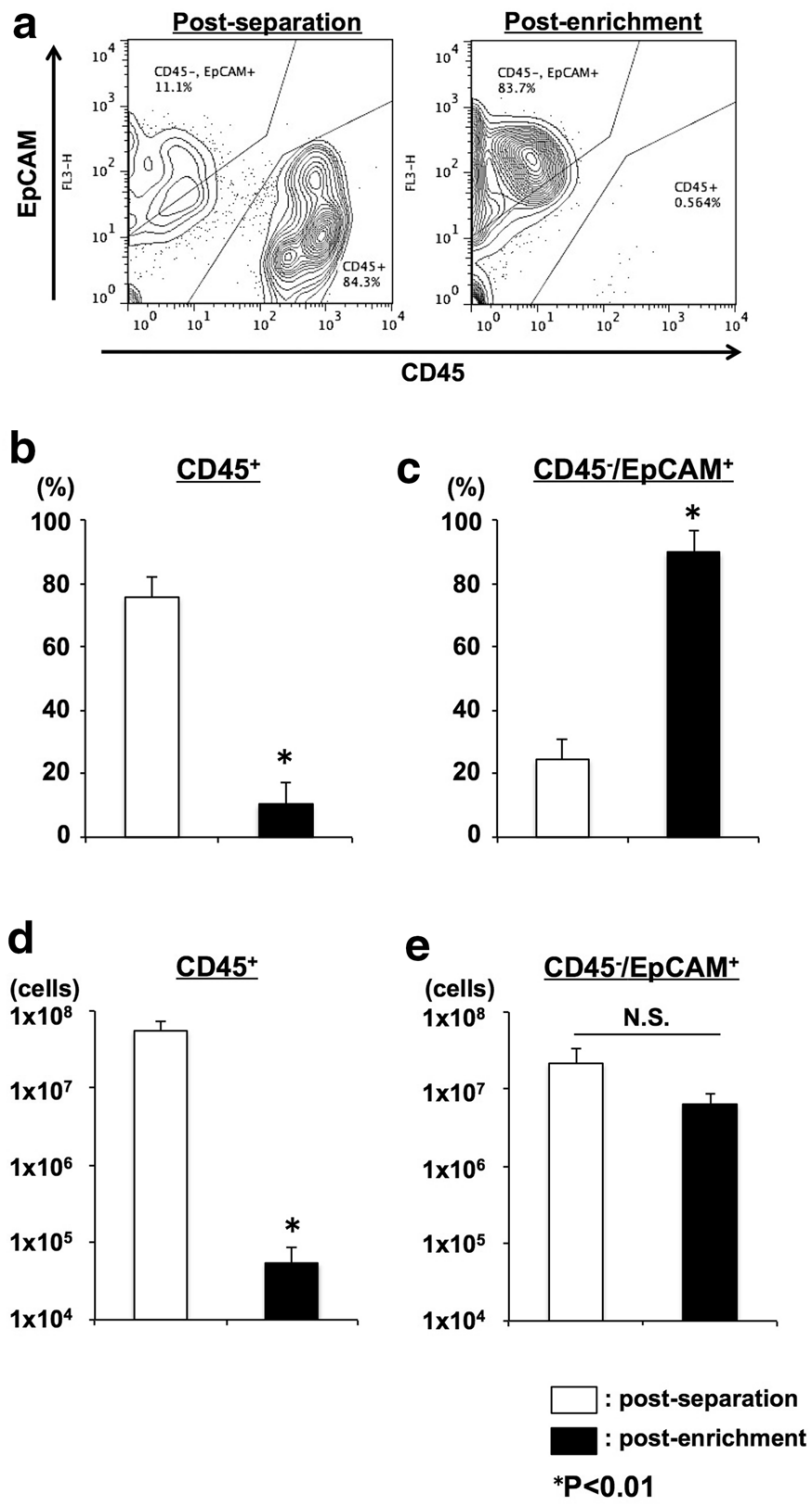
Enrichment of tumor cells for tumor lysate. **a** A typical dotplot pattern of post-separation and post-enrichment cells. Cells were stained with monoclonal antibodies and were defined as CD45^+^CD326^−^; the tumor cells were CD45^−^CD326^+^. **b** The frequencies of CD45^+^ cells (WBC). **c** The frequencies of CD45^−^/EpCAM^+^ cells (tumor cells). **d** The cell number of CD45^+^ cells (WBC). **e** The cell number of CD45^−^/EpCAM^+^ cells (tumor cells)

We analyzed the constitution of the collected cells using CD45 (WBCs) and CD326 (tumor cells) as cell markers. CD326 (epithelial cell adhesion molecule; EpCAM) was originally the epithelial cell marker, and it has been reported to be expressed by approx. 87–100 % of the main ascites-causing carcinomas (Passebosc-Faure et al. 2005; De Angelis et al. 1992; Diaz-Arias et al. 1993; Went et al. 2004). In the present study, the separated cells contained a large number of CD45^+^ (leukocyte-specific antigen) cells (32.96–99.44 %) (Fig. 3a, left panel), and we thus attempted to enrich the tumor cells by CD45 depletion. Through this enrichment step, CD45^+^ cells were removed and CD45^−^/EpCAM^+^ tumor cells were enriched (Fig. 3a, right panel). Although measurable debris has been detected (fluorescence intensity <2×10^0^), the following step (lysate extraction) was carried out without difficulties.

The mean CD45^+^ and CD45^−^/EpCAM^+^ contents are shown in Fig. 3b,c. After the enrichment step, the mean yield CD45^−^/EpCAM^+^ cell number [mean yield total cell number × CD45^−^/EpCAM^+^ (%)] was 6.34 × 10^6^ cells (range 4.74 × 10^4^–2.53 × 10^7^ cells) (Fig. 3d). In contrast, the mean yield CD45 ^+^ cell number [mean yield total cell number × CD45 ^+^ (%)] was 5.40 ×10^4^ cells (range 3.30 × 10^3^–4.19 ×10^5^ cells) (Fig. 3e). Summary of KM-CART and total cells and tumor cells acquired after enrichment step is shown in Table 2.

**Table 2:** Summary of KM-CART and total cells and tumor cells acquired after enrichment step

Patient ID	Volume of ascites (mL)	Volume of lavage fluid (mL)	Total cell No. (×10^6^)	CD45^−^/EpCAM^+^ cell No. (×10^6^)
1	3750	800	18.6	18.5
2	5900	3000	17.8	17.8
3	4900	1700	0.07	0.06
4	6400	3150	25.3	25.3
5	5100	2560	2.10	2.09
6	8050	6800	6.72	6.70
7	4500	1700	0.78	0.77
8	2800	1700	0.32	0.28
9	7000	2630	0.47	0.47
10	3800	1580	1.60	1.59
11	11,500	1000	8.64	8.56
12	9300	6400	0.13	0.12
13	8500	1600	0.64	0.61

**Fig. 4:**
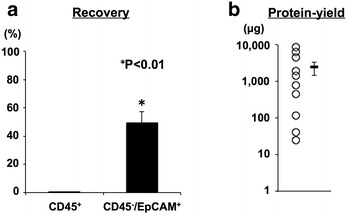
Protein contents extraction from tumor cells. Protein contents were extracted from enriched tumor cells by the freeze and thaw method. **a** Recovery efficiency of WBC (CD45^+^) and tumor cells (CD45^−^/EpCAM^+^). **b** Protein yield from total ascites

The recovery efficiency of CD45^+^ cells was 0.30 % (0.01–2.67 %), and that of CD45^−^/EpCAM^+^ cells was 49.07 % (7.00–94.90 %) (Fig. 4a). Finally, the tumor lysate was extracted from purified CD45^−^/EpCAM^+^ tumor cells. The mean yield amount of tumor lysate was 2416.87 μg (range 25–8743 μg) (Fig. 4b).

## Discussion

Latifi A et al. reported that the tumor cells in the ascites of the patients had an antigenic phenotype that was identical to that of their solid tumor counterpart (Latifi et al. 2012). Provencher et al. also reported that tumor cells from malignant tissue and ascetic fluid resembled the antigenic phenotype (Provencher et al. 1993). Thus, the tumor cell lysate from ascites could be an ideal source with which to produce tumor-specific vaccines.

The results of the present study showed that highly purified tumor cells and tumor cell-associated protein could be obtained from KM-CART lavage fluid in a simple manner. Filtration (Hirte et al. 1992), peptide-conjugated nanoparticles (Scarberry et al. 2010), density gradient centrifugation (Hamburger et al. 1985) and magnetic beads (Barker et al. 2001; Chan et al. 2007) have been used to separate tumor cells from malignant ascites. Among these methods, magnetic cell separation might be useful for the enrichment of tumor cells because of the high purity and high yield it can provide. However, due to the solidification of the content during the shipping of the ascites, the magnetic separation column can become clogged. Cold-induced precipitation of cryogel from plasma has been reported by Morrison et al. (Morrison et al. 1948). Cryogel is a physical gel formed by the heterophilic aggregation of fibronectin, fibrinogen and heparin (Miyamoto et al. 2001). In our KM-CART method, the solid contents containing tumor cells in the ascites were treated with collagenase, and thus the mAb-conjugated magnetic beads’ access to target cells might be improved, efficiently separating the cells. The clinical feasibility of our simple preparation method for generating tumor lysate should therefore be examined in clinical studies of DC vaccines.
